# Supplementary material for: Caffeine induces age-dependent increases in brain complexity and criticality during sleep
Source: Commun Biol. 2025 Apr 30;8:685. doi: 10.1038/s42003-025-08090-z (PMC12044076; doi:10.1038/s42003-025-08090-z)
Supplement: Supplementary file 2 — Supplementary Material [file 42003_2025_8090_MOESM2_ESM.pdf]

Supplementary Material

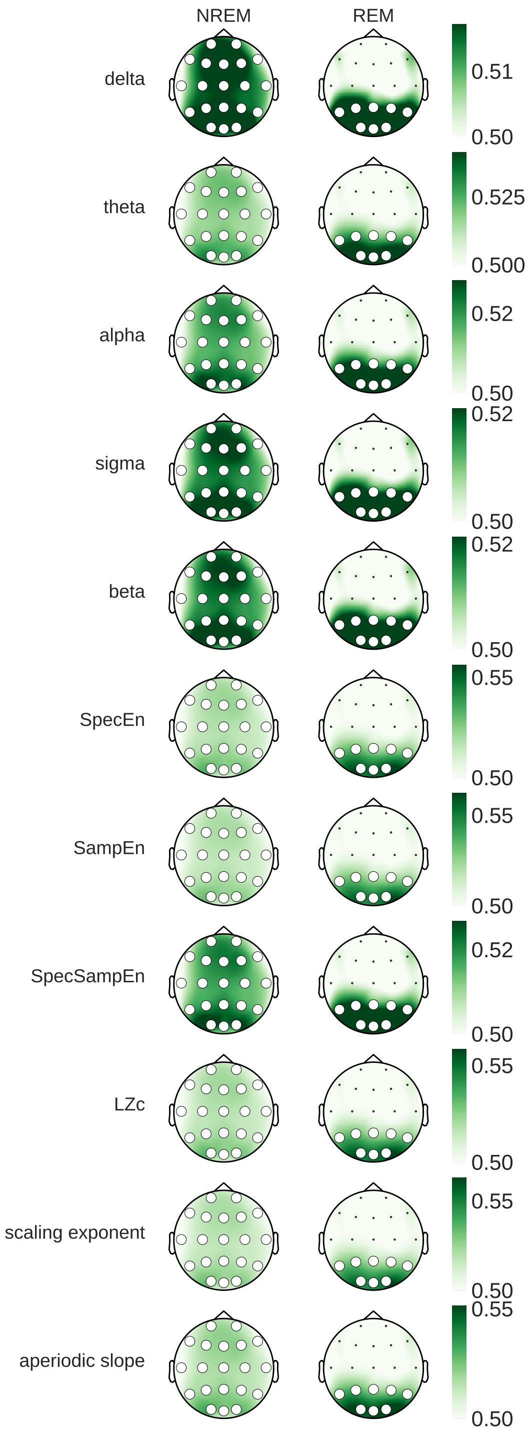

Fig S1. Single-epoch Classification. Topographical maps showing LDA classification scores of single-feature, single-channel classification on 20s epochs (instead of averaged features). Due to class imbalance, classification performance is measured using the balanced accuracy (BAcc) metric, resulting in a chance-level score of 0.5. Statistical significance was evaluated using permutation tests and corrected for multiple comparisons using maximum statistics (grey: $p<0.05$, white: $p<0.01$).

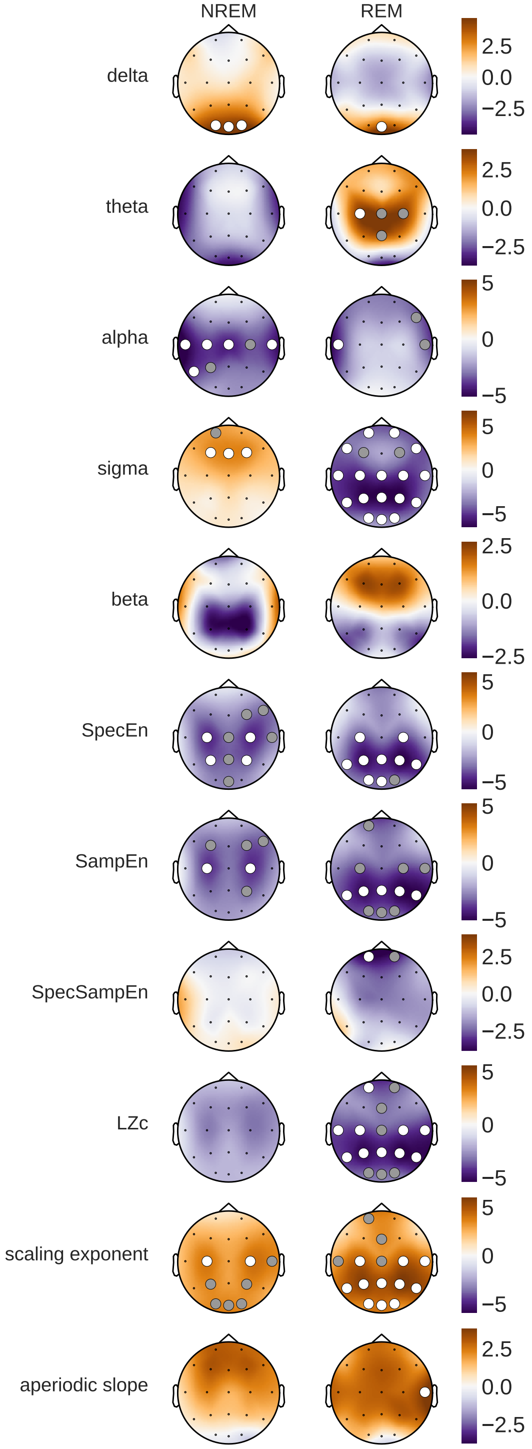

Fig S2. Age-related Baseline Effects (placebo-nights only). t-values from independent T-tests between the young (20-27 years) vs. middle-aged (41-58 years) group on data taken only from the placebo condition. Orange corresponds to larger values in the young group while purple indicates larger values in the middle-aged group. We calculated p-values from the test statistics and corrected for multiple comparisons using the Bonferroni method (grey: p<0.05, white: p<0.01).

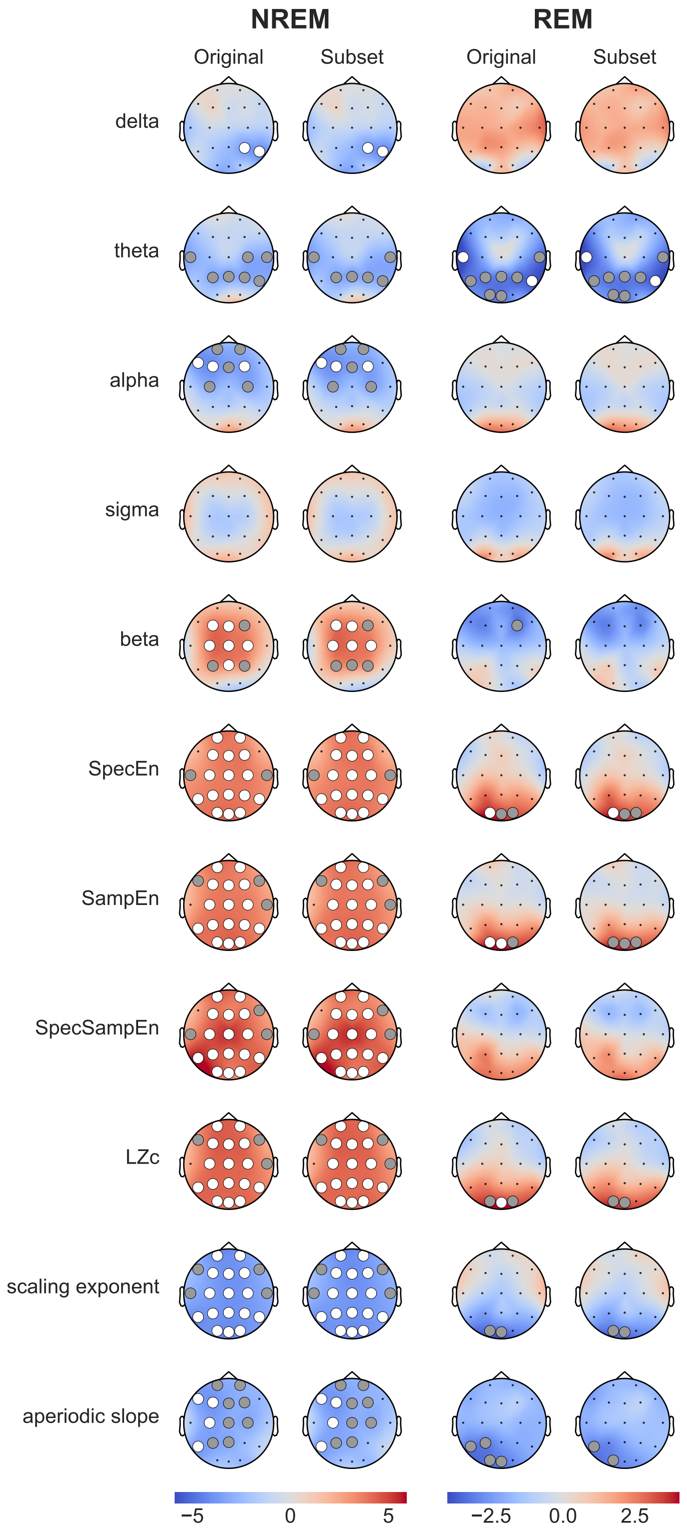

Fig S3. Equating Sleep Duration. t-values comparing statistical findings with and without equating the number of epochs between caffeine and placebo conditions. The "Subset" columns show the results of the control analysis for mitigating sleep duration as a potential confound. Data in the "Original" columns is the same as presented in Figures **??** and **??**. Grey dots represent significance at p<0.05, white dots indicate p<0.01, all corrected for multiple comparisons using maximum statistics.

Parameter	Values
#estimators	{75, 100, 150, 200}
max. depth	{inf, 50, 75}
criterion	{gini, entropy}
max. features	{sqrt, log2}
min. samples per leaf	{1, 5, 10}
bootstrap	{true, false}

Table S1. Grid search parameter selection. Selection of hyperparameters and corresponding value options for the grid-search optimization of the random forest (RF). The values correspond to the hyperparameter options as implemented in scikit-learn.

**NREM**			**REM**		
feature	electrode	importance	feature	electrode	importance
LZc	P4	0.018	theta	T3	0.014
SpecSampEn	C3	0.018	scaling exponent	O1	0.013
SpecSampEn	Cz	0.018	SpecEn	O1	0.012
LZc	F3	0.018	SpecEn	T5	0.011
LZc	P3	0.016	theta	T6	0.011
LZc	Cz	0.016	theta	T4	0.010
LZc	F4	0.015	sigma	C3	0.010
LZc	C3	0.015	aperiodic slope	P3	0.010
LZc	Pz	0.014	scaling exponent	O2	0.010
LZc	Fz	0.014	theta	T5	0.010

Table S2. Feature importance scores. Ten most important combinations of feature and electrode determined by the random forests' feature importance in descending order. The models were trained and evaluated separately for each sleep stage (NREM and REM).

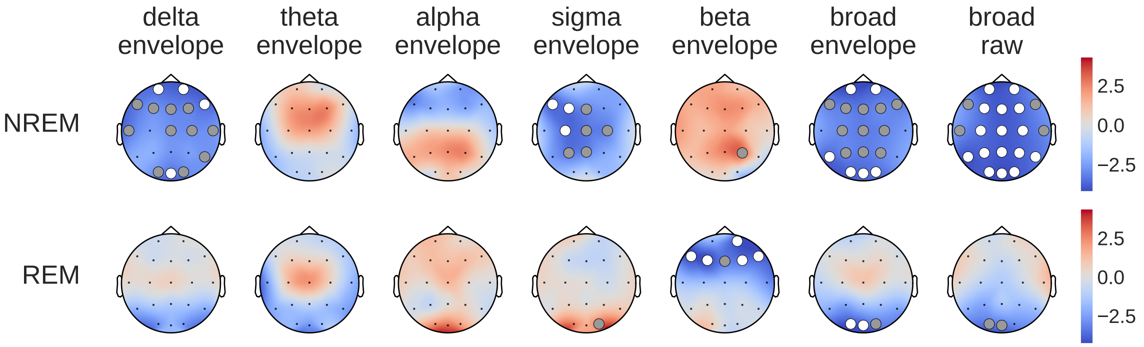

Fig S4. DFA on envelope versus raw. t-values comparing statistical findings of detrended fluctuation analysis (DFA) applied to the signal's envelope in contrast to the raw data. We investigate the effect of narrow-band filtering before computing envelopes and compare to broadband DFA. Grey dots represent significance at $p<0.05$, white dots indicate $p<0.01$, all corrected for multiple comparisons using maximum statistics.

Feature	NREM	REM
delta	-0.633**	0.421
theta	-0.504*	-0.637**
alpha	-0.641**	0.306
sigma	-0.264	-0.296
beta	0.641**	-0.512*
SpecEn	0.668**	0.603**
SampEn	0.696**	0.578**
SpecSampEn	0.933**	0.402
LZc	0.712**	0.589**
scaling exponent	-0.668**	-0.531*
aperiodic slope	-0.612**	-0.512*

Table S3. Peak effect sizes across features. Cohen's d values with largest absolute values for each feature and sleep stage. Significance levels: $p<0.05$ (*) and $p<0.01$ (**).
