# Supplementary material for: Caffeine induces age-dependent increases in brain complexity and criticality during sleep
Source: Commun Biol. 2025 Apr 30;8:685. doi: 10.1038/s42003-025-08090-z (PMC12044076; doi:10.1038/s42003-025-08090-z)
Supplement: Supplementary file 3 — Reporting Summary [file 42003_2025_8090_MOESM3_ESM.pdf]

Corresponding author(s): Philipp Thölke
Last updated by author(s): Apr 11, 2025

# Reporting Summary

Nature Portfolio wishes to improve the reproducibility of the work that we publish. This form provides structure for consistency and transparency in reporting. For further information on Nature Portfolio policies, see our Editorial Policies and the Editorial Policy Checklist.

## Statistics

For all statistical analyses, confirm that the following items are present in the figure legend, table legend, main text, or Methods section.

| n/a | Confirmed | |
|---|---|---|
| ☐ | ☒ | The exact sample size (*n*) for each experimental group/condition, given as a discrete number and unit of measurement |
| ☐ | ☒ | A statement on whether measurements were taken from distinct samples or whether the same sample was measured repeatedly |
| ☐ | ☒ | The statistical test(s) used AND whether they are one- or two-sided<br>*Only common tests should be described solely by name; describe more complex techniques in the Methods section.* |
| ☐ | ☒ | A description of all covariates tested |
| ☐ | ☒ | A description of any assumptions or corrections, such as tests of normality and adjustment for multiple comparisons |
| ☐ | ☒ | A full description of the statistical parameters including central tendency (e.g. means) or other basic estimates (e.g. regression coefficient) AND variation (e.g. standard deviation) or associated estimates of uncertainty (e.g. confidence intervals) |
| ☐ | ☒ | For null hypothesis testing, the test statistic (e.g. *F*, *t*, *r*) with confidence intervals, effect sizes, degrees of freedom and *P* value noted<br>*Give P values as exact values whenever suitable.* |
| ☒ | ☐ | For Bayesian analysis, information on the choice of priors and Markov chain Monte Carlo settings |
| ☒ | ☐ | For hierarchical and complex designs, identification of the appropriate level for tests and full reporting of outcomes |
| ☐ | ☒ | Estimates of effect sizes (e.g. Cohen's *d*, Pearson's *r*), indicating how they were calculated |

*Our web collection on statistics for biologists contains articles on many of the points above.*

## Software and code

Policy information about availability of computer code

| | |
|---|---|
| Data collection | Harmonie 5.1, Stellate Systems - digitization and recording of EEG data<br>LUNA, Stellate System - sleep scoring |
| Data analysis | All analyses were done with Python 3.8, utilizing the software modules scikit-learn for machine learning, MNE-Python for visualization and, together with statsmodels, statistical analysis. The SciPy stack was used for efficient data structures, mathematical operations and plotting functionalities. A large share of the code was developed inside the Jupyter Notebook and IPython framework. The machine learning analysis was enabled in part by support provided by the Digital Research Alliance of Canada (alliancecan.ca). All code used for analysis and visualization can be found at github.com/dav0dea/caffeine-sleep. |

For manuscripts utilizing custom algorithms or software that are central to the research but not yet described in published literature, software must be made available to editors and reviewers. We strongly encourage code deposition in a community repository (e.g. GitHub). See the Nature Portfolio guidelines for submitting code & software for further information.

## Data

Policy information about availability of data

All manuscripts must include a data availability statement. This statement should provide the following information, where applicable:

- Accession codes, unique identifiers, or web links for publicly available datasets
- A description of any restrictions on data availability
- For clinical datasets or third party data, please ensure that the statement adheres to our policy

EEG data cannot be shared as participant consent for public data sharing was not obtained at the time of collection.

## Research involving human participants, their data, or biological material

Policy information about studies with human participants or human data. See also policy information about sex, gender (identity/presentation), and sexual orientation and race, ethnicity and racism.

| | |
|---|---|
| Reporting on sex and gender | Sex was considered in study design, number of male/female participants were equated (21 males, 19 females). No sex or gender based analysis was performed. |
| Reporting on race, ethnicity, or other socially relevant groupings | No data about race, ethnicity, or other socially relevant groupings were collected. |
| Population characteristics | Age-related analysis was performed in two sub-groups of the population. |
| Recruitment | Participants were recruited based on strict health, sleep, and hormonal criteria, with exclusion of any medical, psychiatric, or sleep-related disturbances confirmed through clinical screening and polysomnography. See section "Data acquisition" for a complete list of exclusion criteria. |
| Ethics oversight | Ethics Committee Hôpital du Sacré-Cœur-de-Montréal |

Note that full information on the approval of the study protocol must also be provided in the manuscript.

# Field-specific reporting

Please select the one below that is the best fit for your research. If you are not sure, read the appropriate sections before making your selection.

☒ Life sciences ☐ Behavioural & social sciences ☐ Ecological, evolutionary & environmental sciences

For a reference copy of the document with all sections, see nature.com/documents/nr-reporting-summary-flat.pdf

# Life sciences study design

All studies must disclose on these points even when the disclosure is negative.

| | |
|---|---|
| Sample size | 40 participants, 2 full night EEG recordings per subject |
| Data exclusions | EMG artifact containing epochs were detected and excluded automatically, further artifacts were excluded by visual inspection |
| Replication | Results were replicated in different configurations of feature extraction and data analysis; results from classical statistical analysis were confirmed by machine learning based analysis |
| Randomization | All participants were recorded under both conditions (caffeine/placebo) in separate recordings, order of conditions was randomized. |
| Blinding | Double-blind cross-over design. |

# Reporting for specific materials, systems and methods

We require information from authors about some types of materials, experimental systems and methods used in many studies. Here, indicate whether each material, system or method listed is relevant to your study. If you are not sure if a list item applies to your research, read the appropriate section before selecting a response.

## Materials & experimental systems

| n/a | Involved in the study |
|---|---|
| ☒ | ☐ Antibodies |
| ☒ | ☐ Eukaryotic cell lines |
| ☒ | ☐ Palaeontology and archaeology |
| ☒ | ☐ Animals and other organisms |
| ☒ | ☐ Clinical data |
| ☒ | ☐ Dual use research of concern |
| ☒ | ☐ Plants |

## Methods

| n/a | Involved in the study |
|---|---|
| ☒ | ☐ ChIP-seq |
| ☒ | ☐ Flow cytometry |
| ☒ | ☐ MRI-based neuroimaging |

# Plants

| | |
|---|---|
| Seed stocks | *Report on the source of all seed stocks or other plant material used. If applicable, state the seed stock centre and catalogue number. If plant specimens were collected from the field, describe the collection location, date and sampling procedures.* |
| Novel plant genotypes | *Describe the methods by which all novel plant genotypes were produced. This includes those generated by transgenic approaches, gene editing, chemical/radiation-based mutagenesis and hybridization. For transgenic lines, describe the transformation method, the number of independent lines analyzed and the generation upon which experiments were performed. For gene-edited lines, describe the editor used, the endogenous sequence targeted for editing, the targeting guide RNA sequence (if applicable) and how the editor was applied.* |
| Authentication | *Describe any authentication procedures for each seed stock used or novel genotype generated. Describe any experiments used to assess the effect of a mutation and, where applicable, how potential secondary effects (e.g. second site T-DNA insertions, mosiacism, off-target gene editing) were examined.* |
